# Unconventional approaches for chiral resolution

**DOI:** 10.1007/s00216-024-05329-2

**Published:** 2024-05-16

**Authors:** Filippo Malacarne, Sara Grecchi, Malinee Niamlaem, Bartlomiej Bonczak, Gerardo Salinas, Serena Arnaboldi

**Affiliations:** 1 Dip. Di Chimica, Università degli Studi di Milano, Milan, Italy; 2 Université de Bordeaux, CNRS, Bordeaux INP, ISM, UMR 5255, 33607 Pessac, France

**Keywords:** Chiral resolution, CISS effect, Electroassisted methods, Wireless approaches, Chiral selectors

## Abstract

Chirality is a fundamental and ubiquitous property of nature involved in multiple fields of science. In particular, the possible resolution of the enantiomeric forms of a molecule is crucial in the pharmaceutical, food, and agrochemical industries. The search for efficient, broad-spectrum, and yet simple methods for obtaining enantiomerically pure substances is a current challenge. Enantioselective resolution methods rely on an asymmetric environment that allows the two antipodes of a chiral molecule to be distinguished. In addition to enantiomeric separation techniques, such as chromatography and electrophoresis, new promising approaches involving out-of-the-scheme synergistic effects between chiral selectors (CS) and external stimuli are emerging. This Trends article discusses different enantioselective mechanisms triggered by unconventional physicochemical stimuli for the design of *avant-garde* approaches that could offer novel perspectives in the field of chiral resolution.

**Graphical abstract:**

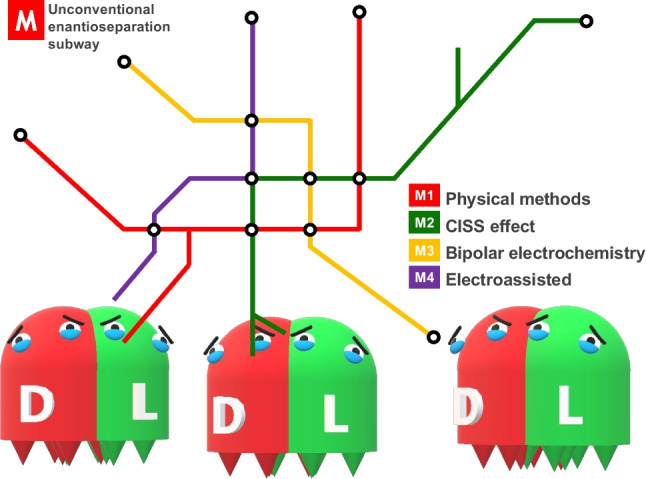

## Introduction

Chirality is the asymmetric property of an object in which two specular, non-superimposable forms, called enantiomers, co-exist [1, 2]. This fundamental feature of nature can be defined over different length scales [3], from the molecular to the micro- and macroscopic environment [4]. Although the two enantiomeric forms exhibit identical physicochemical properties, these differ in the presence of a chiral environment, leading to interactions due to steric effects or diastereoisomeric transition states, thus introducing an energetic difference that allows the recognition and separation of the left- and right-handed enantiomers [5, 6]. In particular, this feature is crucial for biological systems existing in nature in just one configuration and, hence, interacting preferentially with one enantiomer of a chiral substrate. The increasing interest in producing pure enantiomers is not only restricted to the pharmaceutical industry but extends to food and agrochemical production [7, 8]. Furthermore, enantiomeric mixtures produced during asymmetric synthesis require straightforward chiral resolution technologies.

Classic chiral resolution methods include high-performance liquid chromatography (HPLC) [9–12], gas chromatography (GC), supercritical fluid chromatography (SFC) [13], microfluidics techniques such as capillary electrophoresis (CE), capillary electrochromatography (CEC), capillary electrokinetic chromatography (CEKC) [14], and chiral membrane separation [15–17]. In each technique, the possible separation of enantiomers is triggered by the introduction of an asymmetric environment using specific chiral selectors (CSs), e.g., molecular chiral grafted solid particles [18], porous frameworks [19], molecularly imprinted materials [20], π-conjugated polymer films [21], or enantiopure complexing agents [22]. In addition, metal-organic frameworks (MOFs) [23], covalent organic frameworks (COFs) [24], porous organic cages (POCs) [25], and metallacycles [26] have become attractive alternatives as CSs for classic chiral resolution methodologies. The continuous research concerning the design of CSs has expanded over the last few years, stimulating the development of novel and rather unorthodox methods for enantiomeric separation. The present trend article aims to discuss relevant advances in this field, describing more or less complex enantioselective mechanisms triggered by unconventional physicochemical stimuli. However, it is important to highlight that the approaches described here can be considered complementary tools for chiral resolution. In synergy with classic chromatographic and capillary methods, these expand fundamental research and the palette of alternatives for efficient enantioseparation and recognition.

## Unconventional methods for enantioselective separation

As stated above, chromatographic and capillary methods remain the key tools for enantioseparation at the industrial scale. Nonetheless, these traditional methods have evolved to guarantee high sensitivity, high reliability, and robustness for the broadest selection possible of chiral analytes; their main disadvantages still need to be overcome, such as increased consumption of organic solvents and expensive and sensitive instrumentation. However, multiple efforts have been made to develop novel, straightforward alternatives for resolving chiral analytes. From a fundamental point of view, the design of out-of-the-box enantioseparation methods based on alternative physicochemical stimuli can provide a better understanding of the interactions between the antipodes of a chiral molecule and the asymmetric environment in which they are resolved. For example, different theoretical approaches, ranging from simulations of microfluidic flows with variable vorticity [27] or photoinduced drift of chiral molecules [28] to computational modeling of functionalized nanoporous graphene, acting as “gatekeepers” [29, 30], have been studied. In a first order of approximation, these methodologies could either support established methods or inspire the development of entirely new enantioseparating systems. Unlike these theoretical approaches, multiple unconventional, experimental methods for the enantioseparation of chiral molecules based on different external stimuli, e.g., electric or magnetic fields, and rather sophisticated physicochemical interactions have been developed. Due to their novelty, such easy and straightforward chiral resolution methods have opened up new perspectives for analytical applications; however, the possible scale-up to the preparative scale remains challenging.

### Physical methods

These types of separation methods commonly take advantage of the differences in physical properties of the components within a mixture. However, as it is well known, the two enantiomers of a molecule exhibit the same scalar properties; therefore, a chiral environment is required. Although physical methods, based on adsorption, filtration, and crystallization, are well-established approaches, the unconventionality relies on using rather sophisticated CSs or external stimuli to achieve enantioseparation.

**Fig. 1:**
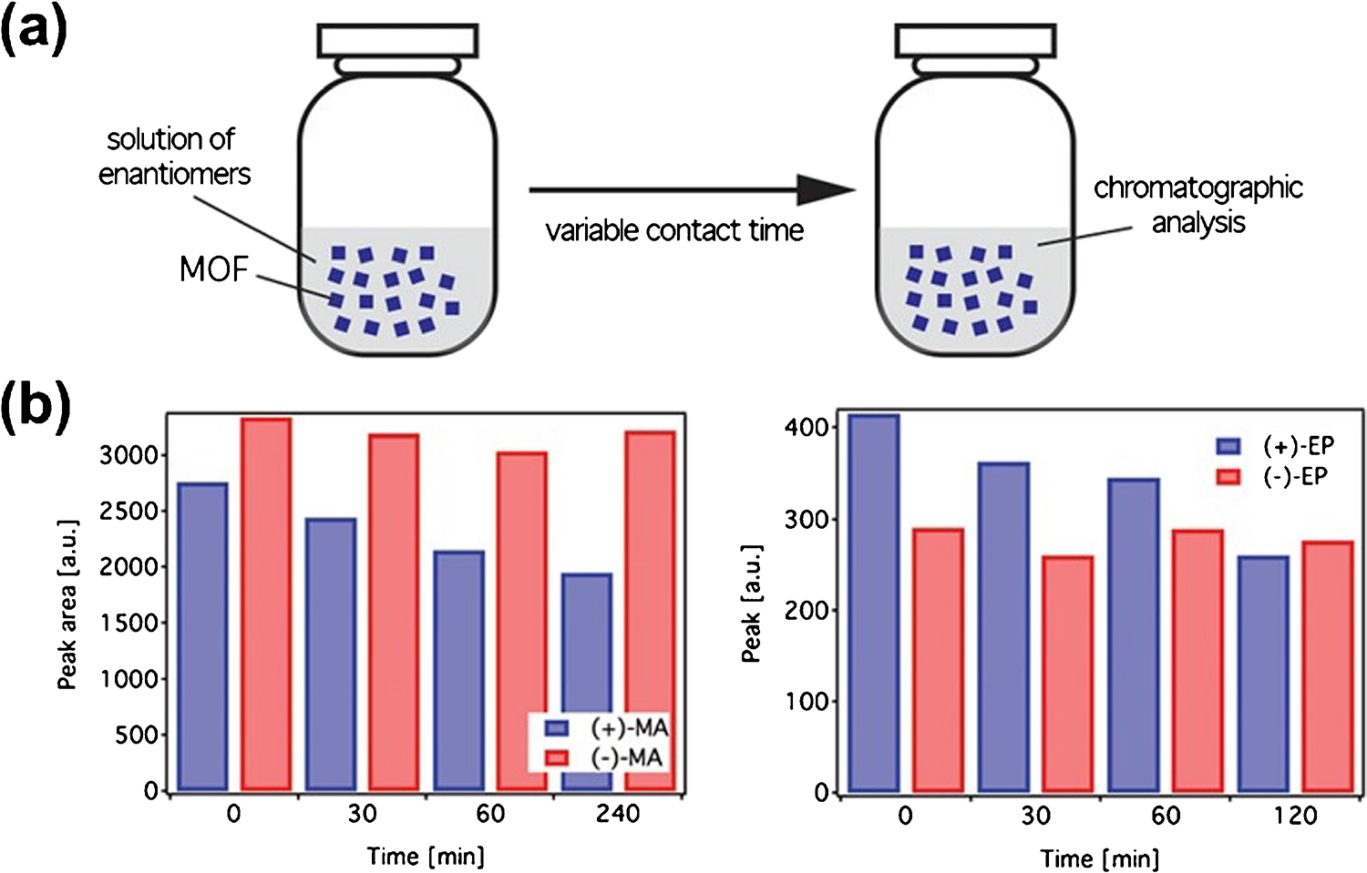
**a** Schematic illustration of the chiral adsorption experimental set-up. **b** Time evolution of the enantioselective recognition of Cu(II) 3D MOF for chiral drugs with contact time for MA (left) and EP (right). Adapted from reference [35]

One of these approaches is based on the selective adsorption of a chiral molecule on a molecularly designed selector, exploiting intermolecular interactions or simply cavity host specificity [31]. For this, only the physical mixing of the selector and the enantiomeric mixture is required for a specific time (Fig. 1a). Due to their structural and physicochemical properties, homochiral MOFs remain the primary type of macromolecules used for this separation [32]. For example, carboxylated-decorated MOF with an intrinsic 3D helical chirality was used for the enantioseparation of (*S*)-1-(1-naphthyl) ethanol with high enantiomeric excess (ee 99%) [33]. Isostructural chiral MOFs bearing dihydroxy groups were used as solid-state host cavities during the efficient adsorption of mixtures of chiral aromatic and aliphatic amines with high enantioselectivity (ee > 80%) [34]. The use of MOFs has been extended to the enantioselective separation of chiral pharmaceutical compounds such as methamphetamine (MA) and ephedrine (EP) [35]. In this work, the chiral Cu(II) 3D MOF based on the tripeptide gly-L-Hys-Gly exhibits host-guest intermolecular interactions with a specific enantiomer simply by fine-tuning the peptide sequence. Thus, the continuous contact between the chiral adsorbent and the target antipode leads to a time-dependent enantioselective separation, in this case of (+)-EP and (+)-MA from a racemic mixture (Fig. 1b). Nonetheless, the possible scale-up to the industrial scale of these approaches remains limited due to the high production costs of these materials and the time-consuming incubation/loading procedures.

**Fig. 2:**
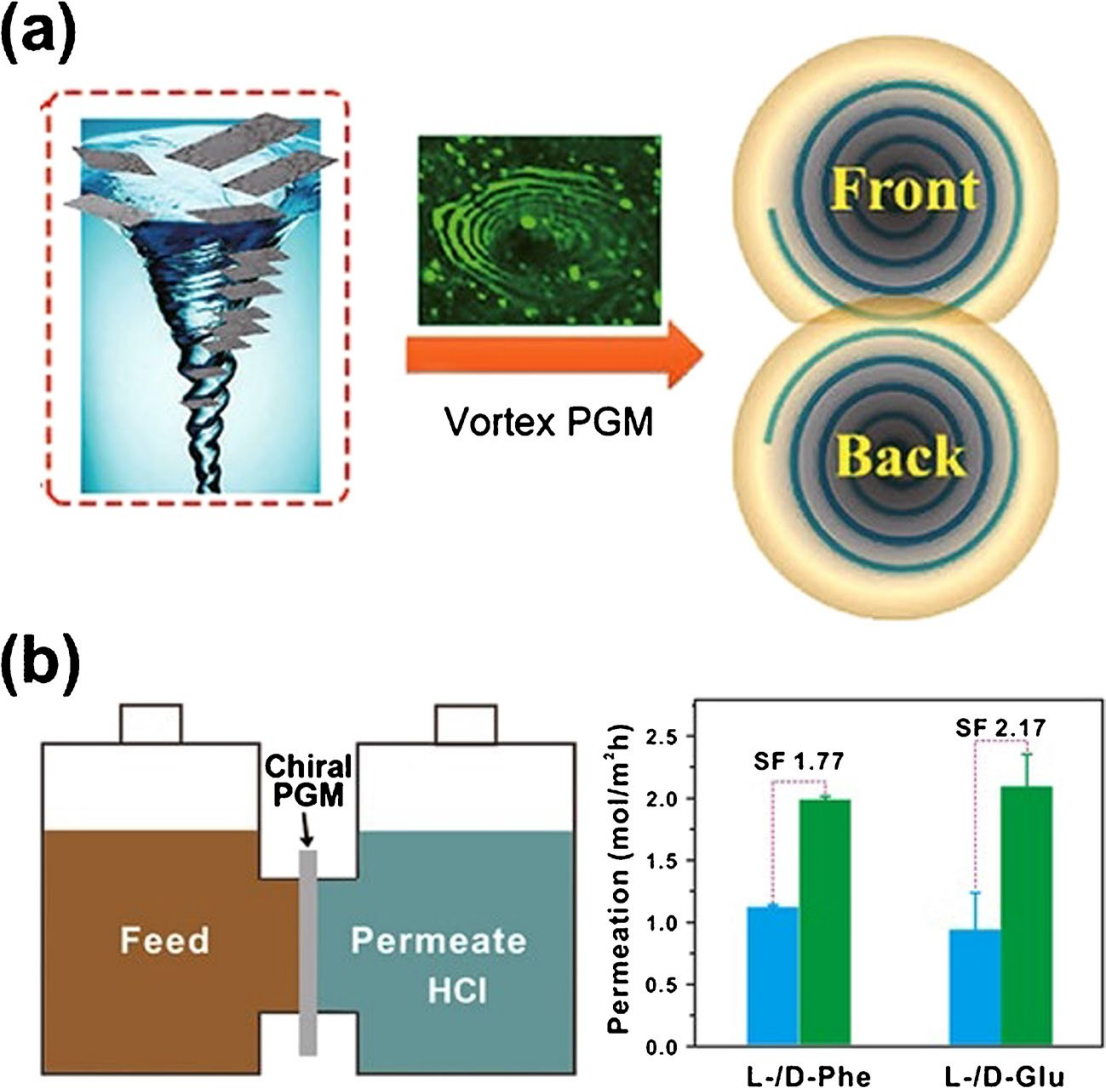
**a** Illustration of preparing chiral porous graphene membranes with a representation of the vortex and the resulting front and back membranes. **b** Schematic illustration of the separation set-up (left) and the separation performance of L-/D-Phe and L-/D-Glu after 48 h (right). Adapted from reference [39]

Recently, chiral selective membranes have gained considerable attention as enantioselective filters. Commonly, this methodology takes advantage of the possible immobilization of chiral selectors along the micro- or nano-cavity. For example, pillar[6]arenes functionalized microchannel membranes were used for the chiral resolution of (*R*)-phenylglycinol [36]. The microchannel was designed by immobilizing chiral Au nanoparticles (NPs) coated with L-phenylalanine-derived pillararene, acting as CS. The functionalized microchannel exhibits an efficient enantioseparation of (*R*)-phenylglycinol (ee > 90%). In an alternative work, micropore membranes containing L-Trp-L-Ala-P6, acting as chiral ligands, were designed using a layer-by-layer self-assembly method [37]. This membrane was used for the enantioselective separation of (*R*)-ibuprofen from racemic mixtures. However, functionalized membranes present relatively small surface areas, which limit the number of recognition sites, resulting in low enantioseparation efficiencies. A promising alternative is the design of more or less unconventional chiral cavities. Kong et al. designed an asymmetric chiral mesoporous nanofiber via stacking and self-assembly methods [38]. The enantioselectivity is based on a charge polarity mechanism on the inner surface of the clockwise or anticlockwise spiral-type shaped fibers. These membranes exhibited outstanding enantioseparation for a series of amino acids with different isoelectric points. With a similar philosophy, Qui et al. developed a chiral porous graphene membrane for the enantioselective separation of amino acids [39]. Such sophisticated material was prepared by mechanically inducing a vortex structure from non-chiral porous graphene (Fig. 2a). The porous graphene was immobilized on an ultrafiltration membrane to produce the chiral porous graphene device during continuous stirring. Good enantioselective separation of amino acids via the classic permeation process was obtained with separation factors above 1.5 (Fig. 2b). Although the separation performance requires further improvement, this work provides light on the transfer of chirality from external physical fields to achiral molecules or materials.

**Fig. 3:**
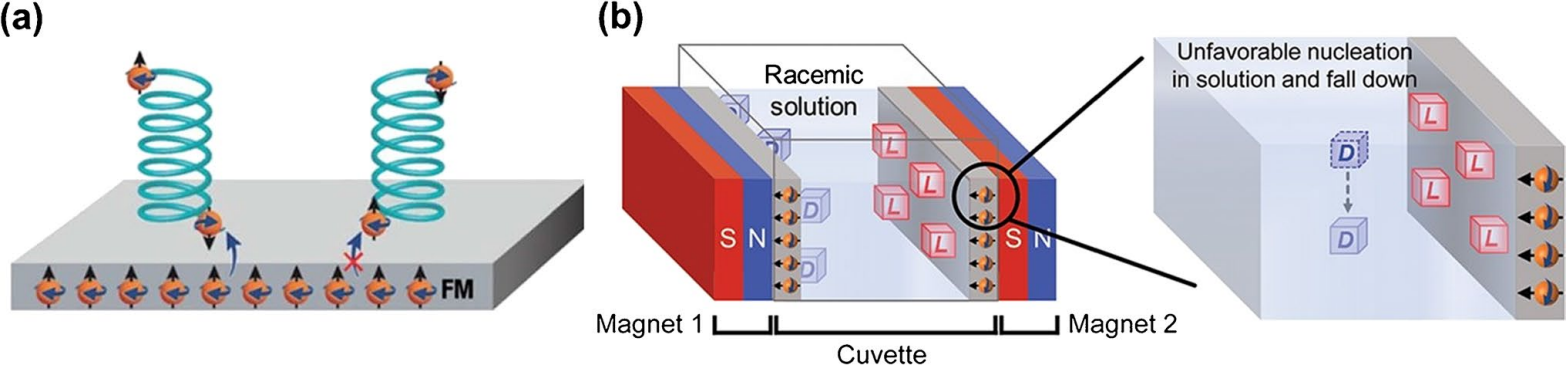
**a** Proposed recognition mechanism based on the chiral-induced spin selectivity. Adapted from reference [50]. **b** Schematic illustration of the experimental set-up used during the simultaneous spin-selective crystallization experiments. Adapted from reference [52]

Another interesting approach is the so-called enantioselective crystallization of chiral molecules [40]. Different methods based on the formation of diastereomeric salts or co-crystallization have been developed and scaled up to the industrial level [41–43]. However, additional purification steps are required due to the strong binding between the antipode of interest and the chiral resolving agent. For this reason, alternative systems have been developed to overcome this issue and minimize further separation procedures. For example, tailor-made polymers for stereoselective crystallization, based on chiral recognition via non-covalent interactions, were designed [44]. These supramolecular additives were used for the enantioselective crystallization of different chiral acids, obtaining good stereoselectivity in yielding crystals with high enantiomeric purities in conglomerates. With a similar philosophy, inherently chiral oligomers of the 2,2′-bis(2,2′-bithiophene-5-yl)-3,3′-bithianaphthene (BT_2_T_4_) have been used as chiral surfaces for the enantioselective crystallization of amino acids [45]. Such oligomers are an unconventional family of π-conjugated polymers that exhibit intrinsic chiral properties since the stereogenic and electroactive elements coincide within the polymeric backbone. Such a fascinating feature allows the induction of favorable or unfavorable diastereomeric interactions between the chiral selector and the enantiomers of a given electroactive analyte, reflected in relatively significant thermodynamic potential differences between the two antipodes [46, 47]. In this work, Flood et al. obtained highly enantiopure crystals of D- or L-ascorbic acid (ee > 90%) on the surface of oligo-(*R*)- or oligo-(*S*)-BT_2_T_4_, respectively, by taking advantage of these favorable or unfavorable diastereomeric interactions, fine-tuning the crystallization rate [45]. Finally, the chiral-induced spin selectivity (CISS) phenomenon was exploited to develop a complete additive-free crystallization approach. The CISS effect relies on the positive or negative coupling of the polarized spin of a chiral molecule with the electron spin of a ferromagnetic material under the effect of a magnetic field orthogonal to the metallic surface (Fig. 3a) [48, 49]. Enantioselective crystallization of asparagine, glutamic acid, and threonine was induced by the spin alignment between the chiral molecule and the ferromagnetic surface [50]. Furthermore, an additional symmetry break was produced by changing the orientation of the external magnetic field. Thus, a selective resolution of the two stereoisomers, as a function of the direction of the magnetic field, was observed. With the same approach, Sasselov et al. used spin-selective crystallization to resolve racemic mixtures of ribo-aminooxazoline (RAO), an RNA precursor, on ferromagnetic surfaces [51]. In this work, crystals with enantiomeric excess between 60 and 100% were obtained. Recently, this spin effect was used for the simultaneous resolution of conglomerates [52]. Fine-tuning the spatial distribution of two ferromagnetic surfaces with opposite magnetization enables the simultaneous spin-selective crystallization on each surface of a different enantiomer (Fig. 3b). However, it is noteworthy to highlight that the enantioselectivity is not directly related to the orientation of the external magnetic field but more especially to the influence of this physical perturbation on the alignment of the spins within the ferromagnetic materials. Nonetheless, such a spin-selective approach exhibits the main advantage of the possible additive-free resolution of a given antipode compared with classic crystallization methods. From an industrial point of view, the use of the above mentioned unconventional stereoselective crystallization systems remains challenging, mainly due to the complex synthesis of the enantioselective polymers or the rather large dimension of the magnets/electromagnets.

### Electroassisted methods

**Fig. 4:**
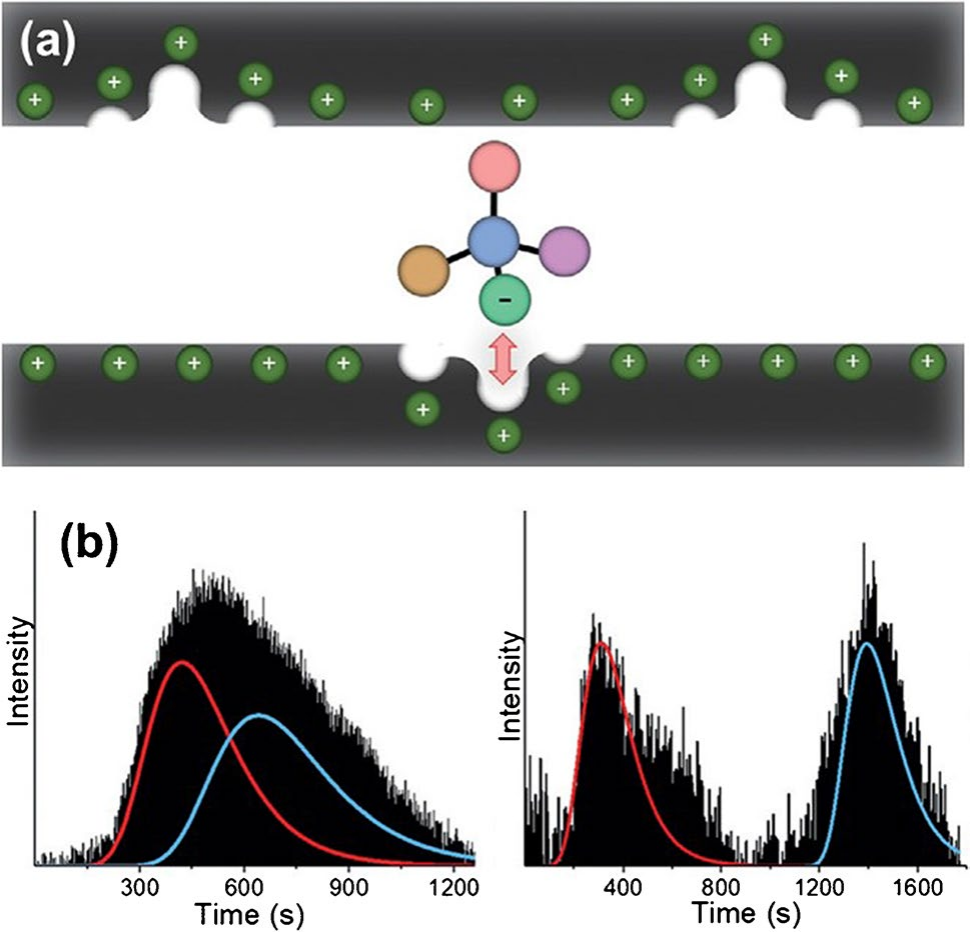
**a** Schematic illustration of the electrostatic interaction of a charged chiral molecule with a cavity in a positively charged mesopore of the microchannel. **b** Electrochromatograms of racemic Tryp solutions with fluorescence detection at the microchannel for L-Tryp imprinted platinum layer in the absence (left) and presence of an applied potential (right 300 mV vs. Ag). Adapted from reference [53]

Although, in theory, these approaches can be misinterpreted as an extension of capillary electrochromatography, the main difference lies within the usage of the applied electric field. In capillary electrochromatography, the electric field triggers an electroosmotic mechanism that allows the motion of the mobile phase. On the contrary, we define an electroassisted approach as a method in which the applied electric field triggers either an adsorption or desorption process of the chiral analyte, allowing the induction of electrostatic interactions between charged analytes and the polarized surface; thus, the electrode acts as the stationary phase (Fig. 4a). Therefore, taking advantage of the synergy between the electrostatic interactions and the chiral cavities is possible by encoding chiral information on the electrode surface. For example, Assavapanumat et al. used a chiral imprinted mesoporous platinum surface for the electroassisted enantioseparation of the two enantiomers of tryptophan (Tryp) and tyrosine [53]. Enantioenriched mixtures were injected into a microchannel decorated with a chiral-encoded porous platinum film by fine-tuning the applied electric field (Fig. 4b). With the same philosophy, hierarchical macroporous chiral MOFs were used for the efficient electroassisted resolution of L- and D-Tryp [54]. Once again, the binding affinity of the designed MOF surfaces was fine-tuned by applying different electric field values, translating to a better resolution of the two antipodes. However, it is essential to highlight that in both cases, a considerable loss of chiral information encoded on the polarized surface was observed when relatively high electric field values were applied. With an opposite philosophy, Santra et al. took advantage of the spin-selective adsorption and electroassisted reductive desorption of chiral thiolated molecules on ferromagnetic surfaces [55]. For this purpose, an arrangement of gold-coated ferromagnetic electrodes, magnetized orthogonally to the metallic substrate, was used as a separation column. Under these conditions, one enantiomer was adsorbed, whereas the opposite one was extracted, depending on the orientation of the magnetic field. In addition, it was possible to collect the adsorbed antipode via the reductive desorption of alkanethiols by applying an electric field. However, with this approach, relatively low ee values for both enantiomers, the extracted and adsorbed ones, were obtained (ee between 40–50%). Despite their rather good efficiency, these electroassisted methods remain proof-of-concept approaches, mainly due to the sophisticated design of the so-called metallic stationary phases, which limits their applicability at the industrial scale.

### Wireless electroassisted approach

**Fig. 5:**
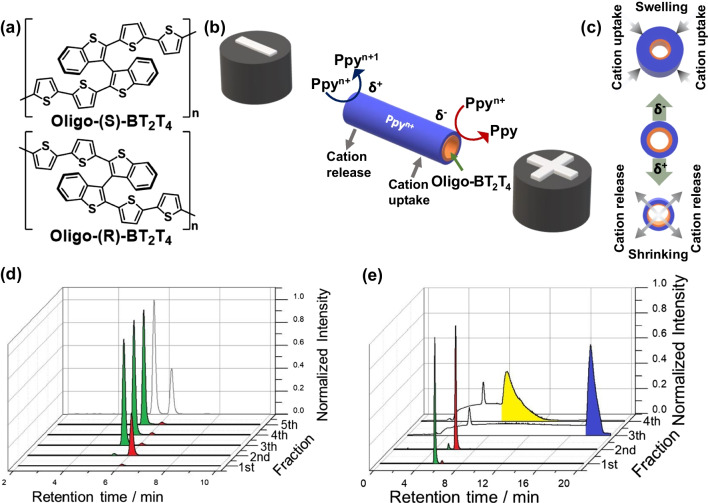
**a** Chemical structures of enantiopure oligo-(*S*)- and oligo-(*R*)-BT_2_T_4_. **b** Illustration of the wireless enantioselective loading/pumping mechanism with a representation of the asymmetric polarization, the associated electrochemical reactions, and the induced cation exchange. **c** The schematic illustration of the electric field-induced asymmetric swelling and shrinking process represents the cation exchange. Adapted from reference [68]. **d** Chromatograms related to the carvone enantioenriched mixture (*S*:*R*) 70:30 extracted from the chiral tube functionalized with the (*S*)-oligomer. **e** Chromatograms of racemates of carvone and N, N-dimethyl-1-ferrocenylethylamine extracted from a chiral tube functionalized with the (*R*)-oligomer. The green and red colors stand for the (*S*)- and (*R*)-carvone, respectively, whereas the yellow and blue colors represent the (*S*)- and (*R*)-N, N-dimethyl-1-ferrocenylethylamine, respectively. Adapted from reference [69]

Although the above-mentioned electroassisted methods are a promising alternative for chiral resolution, a direct electric connection to a power source is still required. In this context, bipolar electrochemistry (BE) has recently gained considerable attention as a wireless alternative to conventional electrochemical methods [56–59]. Briefly, BE is based on the wireless asymmetric polarization of a conductive object, a so-called bipolar electrode (BPE), triggered by applying a high enough external electric field (ε). Under these conditions, a polarization potential difference (*ΔV*) is induced across the BPE, generating cathodic (δ^-^) and anodic (δ^+^) extremities. In the presence of electroactive species, redox reactions take place only when the *ΔV* exceeds the correspondent thermodynamic threshold potential (*ΔV*_*min*_). Thus, it is possible to use this asymmetric reactivity to selectively couple a reaction of interest on one extremity of the BPE with an electrochemical process at the opposite end, acting as a transducer [60–62]. With this philosophy, BE has become an interesting tool for chiral recognition, particularly for the transduction of chirality across different length scales, from the molecular to the macroscopic level [63]. Different and unconventional readouts based on light emission, rotation, and actuation have been used to transduce chiral information in solution [64–67]. Recently, the synergy between wireless actuation and enantioselective recognition was introduced in chiral resolution. This approach takes advantage of two main ingredients: (1) a wireless electromechanical pumping effect and (2) the outstanding enantiorecognition of inherently chiral oligomers. In this work, a chiral tubular hollow electro-pump constituted by a polymeric bilayer was designed [68, 69]. This device was obtained in a two-step approach; at first, the electrooligomerization of the inherently chiral monomer (BT_2_T_4_) (Fig. 5a), followed by the galvanostatic polymerization of pyrrole on the surface of a gold wire, acting as a template. When using this device as BPE and applying a high enough electric field, an electromechanical pumping effect is induced [70], thanks to the asymmetrical polarization along the tube that triggers the oxidation and reduction of doped polypyrrole (Ppy^n+^) at the δ^+^ and δ^-^ extremities, respectively (Fig. 5b). It is well established that such redox reactions are accompanied by an ionic exchange to keep electroneutrality, hence producing swelling and shrinking of the inner diameter at the cathodic and anodic extremities, respectively (Fig. 5c). This asymmetric change of the inner diameter of the tube results in a slow and unidirectional pumping effect according to the Bernoulli principle. In addition, the enantioselectivity is based on favorable or unfavorable diastereomeric interactions between the inherently chiral oligomer, oligo-BT_2_T_4_, and the chiral probe. As a first proof-of-concept, the enantioselective electromechanical pump mimics chiral columns generally used in HPLC, allowing the selective loading and separation of different chiral analytes injected as pure enantiomers or in racemic form [68]. The presence of the enantiopure oligo-BT_2_T_4_ affects the retention time of the chiral analyte injected inside the modified Ppy tube; thus, for favorable diastereomeric interaction, the chiral probe remains trapped inside the tube, whereas for unfavorable interaction, the analyte is expelled almost immediately. Furthermore, these devices were used for the highly efficient enantioseparation of mixtures of racemates and unbalanced samples containing two chiral analytes with uncorrelated chemical structures [69]. The HPLC analyses of the collected fractions corroborate the outstanding enantioseparation, obtaining samples with high enantiomeric purity (ee > 90%) (Fig. 5d and e). These micropumps present advantages from conventional and unconventional approaches, such as their wireless feature, easy and straightforward design, low cost, and relatively short separation times. In addition, it is possible to assume that fine-tuning the dimension of the pumps can expand this methodology to the preparative scale; however, due to the mechanical limitations of the conducting polymer, a real industrial application is still challenging.

## Outlook

The increasing interest in chirality as a field of science is boosting the development of new methods to separate enantiomers and recover optically pure compounds. Traditional chiral resolution techniques, such as chromatography and microfluidic methods, remain widely used. However, the interest in discovering and developing innovative chiral selectors and separation mechanisms is rapidly evolving. In addition, novel unorthodox physical resolution methods with interesting and out-of-the-scheme approaches were investigated to find new, straightforward, and accessible enantioseparation protocols. Such methods can be divided into those implying the selective adsorption of chiral molecules, primarily through the unconventional design of chiral cavities on a molecularly designed selector, and those inducing enantioselective crystallization, developed to minimize additional purification steps typical of industrial scale methods, either by exploiting tailor-made polymers or entirely without additives, through CISS-based applications. Finally, the electroassisted methods differ substantially from capillary electrophoresis and capillary electrochromatography since the electric field triggers the adsorption or desorption of analytes from a polarized surface encoded with chiral information. Although promising, their performances can be further improved thanks to bipolar electrochemistry, which avoids the direct electrical connection of the active components of the devices to a power source. Exploiting the synergy between the wireless electromechanical pumping effect of Ppy and the excellent enantiorecognition properties of oligo-BT_2_T_4_, soft tubular devices have been designed to efficiently separate mixtures of enantiomers, even with uncorrelated chemical structures. Enantiomer separation is a hot research topic, and even though significant steps have been made due to the development of innovative materials and methods, there are still substantial opportunities and challenges in this field. The promising examples of unconventional methods for chiral resolution presented here are still at an early stage of development, and much more work will be required to reach macroscale applications.

Nonetheless, aiming to be a complementary tool to classic separation methods, multiple efforts are underway to improve the separation efficiency and decrease the analysis time of these sophisticated approaches. However, the importance of exploring these new methods lies in meeting the needs of the ever-growing chemical industry and supporting the development of emerging technologies based on chiral materials. We envision that significant progress in this area is ahead and that its impact could extend to multiple fields of science, including chemistry, biology, material science, and physics.
